# Accuracy of fully guided orthodontic mini-implant placement evaluated by cone-beam computed tomography: a study involving human cadaver heads

**DOI:** 10.1007/s00784-020-03436-9

**Published:** 2020-07-01

**Authors:** Kristian Kniha, Maximilian Brandt, Anna Bock, Ali Modabber, Andreas Prescher, Frank Hölzle, Golamreza Danesh, Stephan Christian Möhlhenrich

**Affiliations:** 1 Department of Oral and Maxillofacial Surgery, University Hospital of Aachen, Pauwelsstraße 30, 52074 Aachen, Germany; 2 Private practice for Orthodontics, Blumenstraße 29, 73728 Esslingen, Germany; 3 Medical Faculty of RWTH-Aachen, Institute of Molecular and Cellular Anatomy, Pauwelsstraße 30, 52074 Aachen, Germany; 4 Department of Orthodontics, University of Witten/Herdecke, Alfred-Herrhausen Str. 44, 58455 Witten, Germany

**Keywords:** Template, Guided surgery, Dental implant, Accuracy

## Abstract

**Objectives:**

The aim of this study was to evaluate the accuracy of fully guided orthodontic mini-implant (OMI) placements supported by tooth- (TBGs) or gingiva-borne silicone guides (GBGs) based on virtually superimposed lateral cephalograms on virtual plaster models.

**Materials and methods:**

Lateral cephalograms and corresponding plaster models were virtually superimposed for the planning of OMI positions; fully guided TBGs and GBGs were fabricated (each, *n* = 10). A total of 40 OMIs were inserted in a paramedian position into the palate of 20 human cadavers. Postoperative cone-beam computer tomographies (CBCTs) were carried out, and an accuracy evaluation was performed by comparing preoperative planning models and postoperative CBCTs. Deviations of the axis, tip, centre of the shoulder and vertical position of each of the implants were evaluated. Furthermore, the transfer accuracy measured by postoperative CBCT scans were compared with the accuracy determined using an intraoral scanner.

**Results:**

A significant deviation between TBGs (2.81° SD 2.69) and GBGs (6.22° SD 4.26) regarding implant angulation was evaluated (*p* = 0.005). Implant tip and implant shoulder deviations revealed no statistical differences between the guides. Accuracy values of oral scans regarding vertical deviations were significantly more inaccurate when compared with CBCTs (*p* < 0.001).

**Conclusions:**

The accuracy of an OMI position can be significantly increased by using a guide extension over the teeth. Vertical implant positions presented the lowest deviations. Postoperative oral scans and CBCTs represent diverging accuracy measurements when compared with virtual planning.

**Clinical relevance:**

Users must keep in mind that despite virtual planning deviations, inaccuracies of a few millimetres may occur.

## Introduction

A report on mini-implants for orthodontic anchoring was first published by Gainsforth and Higley in 1945 [1]. The authors used Vitallium screws in an animal study to explore the possibilities of skeletal anchoring. However, these implants showed high loss rates. Further experiments followed and included intermediate steps involving items such as endosseous implants, osteosynthesis screws and conventional dental implants; in modern times, orthodontic mini-implants (OMIs) are used. The term was introduced by Kanomi in 1997 [2].

In recent years, skeletal anchoring with OMIs has been successful due to its advantages with low patient compliance [2–5]. Mini-implants can be placed in the alveolar process between the roots, in the retromolar region or in the anterior palate [6]. Care must be taken at all times to be sure that no surrounding anatomical structures get damaged [7]. Interradicular insertion involves the risk of root damage [8]. Skeletal anchorages in the lower jaw, such as with screws used in intermaxillary splinting, can also damage the mental nerve. Mini-implants exhibit less surgical invasiveness compared with procedures involving invasive osteosynthesis plates [9, 10].

However, the main use of mini-implants is in the upper jaw during orthodontic treatment. Placement on the palate is considered comparatively safe but they should be anchored in cortical structures without perforating the maxillary sinus or nasal floor [11]. A deviating implant position may lead to chronic persistent sinus inflammation. In order to minimise the risk of tooth injury and implant tipping or loss, exact implant positioning is essential for successful treatment.

In a previous investigation, the authors investigated the transfer accuracy of OMI placement at the anterior palate depending on whether tooth-borne guide (TBG) or gingiva-borne guide (GBG) supports were planned on virtual plaster models superimposed with corresponding lateral cephalograms in the oral cavity [12]. The measurements based on postoperative intraoral scans were supported by scanbodies to determine each of the final OMI positions, which were compared with preoperative planning models using automatic surface registration based on an iterative closest-point algorithm. It was found that, depending on direction and guide extension, deviation varied for linear measurements between 0.88 mm (SD 0.46) and 2.34 mm (SD 0.74) and angular measurements between 3.60° (SD 2.89) and 6.46° (SD 5.5). These measurements focused deviations on the oral part of the implant, which is of interest for receiving an orthodontic appliance. However, the intra-bony accuracy is also of concern due the possibility of damaging surrounding anatomical structures.

Therefore, the primary aim of this study was to retrospectively investigate the previously published cadaveric data to evaluate the precision of fully guided OMI placement based on TBGs or GBGs, comparing virtual planning models and corresponding postoperative cone-beam computed tomography (CBCT) scans. The secondary aim was to compare the transfer accuracy measured by postoperative CBCT scans with the accuracy determined using an intraoral scanner combined with scanbodies, as noted in the previously published investigation [12].

## Materials and methods

According to the ethical approval given by the Ethics Committee of the Medical Faculty of the RWTH Aachen, Germany (EK 219/16), institutional approval by the Institute of Molecular and Cellular Anatomy of the University Hospital of the RWTH Aachen, Germany, was obtained. During each of their lifetimes, the donors on which this study is based provided permission for their bodies to be used for research and education. The used heads were fresh, which means they were immediately frozen after death without any fixation solutions; they were later thawed for scientific purposes.

Exclusion criteria included the presence of dental arches with more than two missing teeth per quadrant, gaps larger than one missing tooth, clinical signs of significant atrophy, and the absence of first upper premolars.

Two orthodontic mini-implants (2 × 10 mm, OrthoLox, Promedia Medizintechnik Ahnfeldt GmbH, Siegen, Germany) were placed fully guided without predrilling, paramedian in the anterior palate of 20 human cadaver heads (14 males and 6 females; mean age 71 years, range 66 to 83 years). The body donors were related to a body donor group from a study that was previously published; this study represents an ongoing evaluation [12]. In this investigation, the levels of accuracy for TBG (*n* = 10) and GBG (*n* = 10) supports were evaluated by comparing the planned positions of orthodontic mini-implants and real postoperative positions in the CBCT scans.

### Template fabrication and implantation

**Fig. 1:**
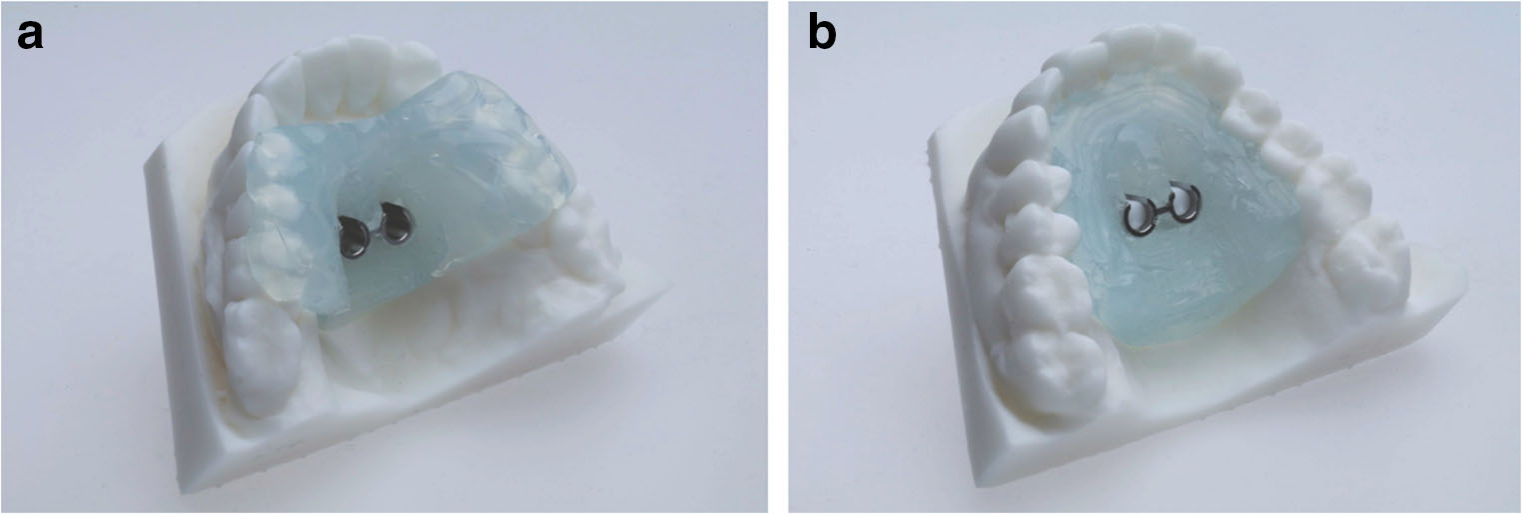
Three-dimensionally printed working models with **a** tooth-borne guide and **b** gingiva-borne guide

For virtual planning, lateral cephalograms (Orthophos SL 2D, Dentsply Sirona, York, Pennsylvania, USA; 73 kV, 15 mAs, effective radiation 9.2 s) were taken. Therefore, the heads were aligned with optical localizers to the midsagittal plane and Frankfurt horizontal plane to ensure symmetry. Cast models were prepared based on impressions using Impregum Penta (3M ESPE, Neuss, Germany), according to the manufacturer’s instructions. This called for automatic mixing in the corresponding Pentamix device and taking the impression material into the oral cavity for 7 min to ensure complete setting. All impressions were previously produced with super-hard plaster (Alpenrock, Amann Girrbach, Koblach, Austria) rotated in a vacuum mixer. To ensure that final hardness had been reached, the models were digitally transferred after 24 h using a 3D model scanner (orthoX scan, Dentaurum, Ispringen, Germany) that provided an average accuracy scanning of 0.5 mm [13]. Lateral cephalograms and corresponding models were matched by software support (TAD match, OnyxCeph, Image Instruments GmbH, Chemnitz, Germany) and used for planning of the appropriate position, as previously described [12]. The templates were produced on the working model that was manufactured by a 3D printer (Form 2, Formlabs, Somerville, MA, USA). The heads were randomly allocated into either tooth- or gingiva-borne groups. Finally, fully guided templates were manufactured with a two-component silicone (Transpasil, Kaniedenta GmbH & Co. KG, Herford, Germany) (Fig. 1a, b). The templates were incorporated into the maxilla, and the precise fit was visually and manually controlled before and during surgery. In all cases, implantation was performed by one experienced surgeon with one assistant taking care of the template position. Placement was performed without predrilling using a contra-angle handpiece drive (Prosthodontic implant driver, W&H, Bürmoos, Austria) with a speed of 25 rpm and an adjustable torque control with a maximum of 40 Ncm. The insertion stopped automatically after the mini-implant reached the final depth by separation of the implant from the insertion aid.

### Accuracy measurements

**Fig. 2:**
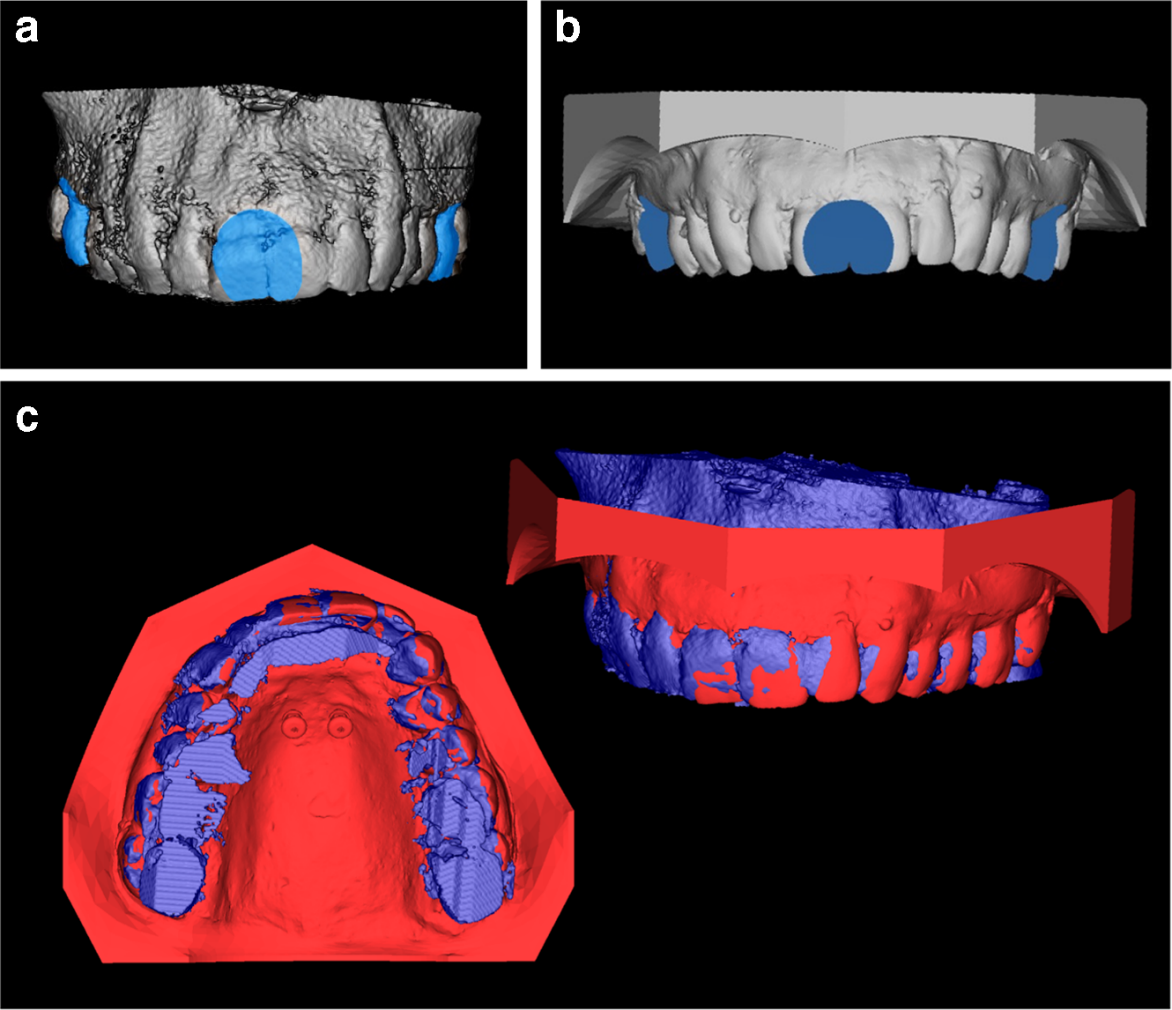
**a** At least three matching references spread over the dental arch were chosen. **b** Additionally, the corresponding matching references were marked on the cast model. **c** Matching of the radiographic file and cast model data using coDiagnostiXTM software

**Fig. 3:**
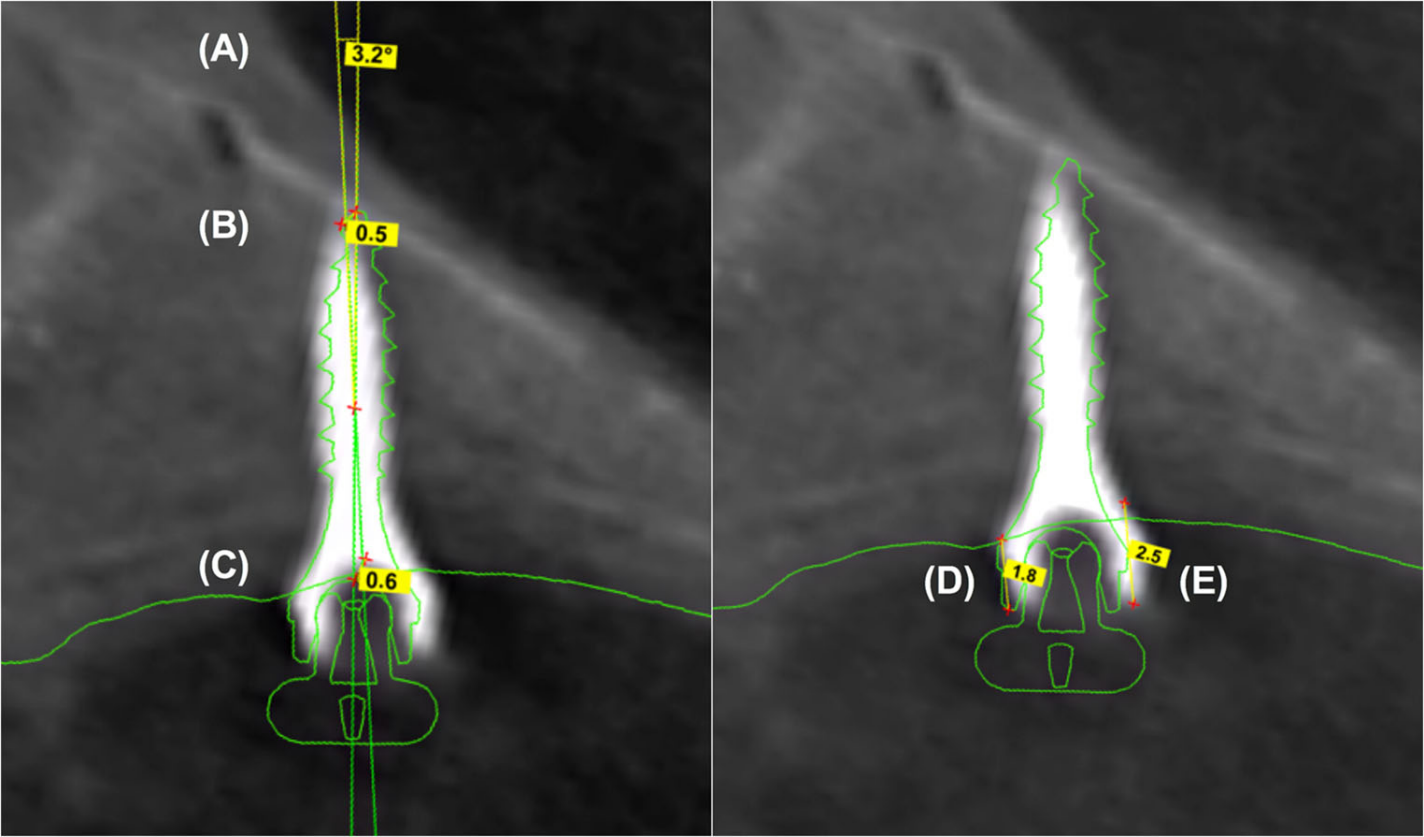
Three-dimensional measurements between the superpositioned files of the deviations of the implant axis (**a**), implant tip (**b**) and implant shoulder (**c**). Evaluation of the distance between the vertical implant position of the anterior (**d**) and posterior implant shoulder (**e**) to the contact of the gingiva at the implant. Measurements of the anterior and posterior vertical implant position were performed twice: during preoperative planning (**d**) and during postoperative CBCT scans (**e**)

Postoperative CBCT scans (Galileos, Sirona, Bensheim, Germany; 98 kV, 25 mAs, effective radiation time 5 s, FOV: diameter 215 mm, voxel size 160 μm) were performed after the heads were aligned with optical localizers to the midsagittal plane and Frankfurt horizontal plane to ensure symmetry. Then, the scans were transformed into the Digital Imaging and Communications in Medicine (DICOM) format and imported with the virtual cast models into the coDiagnostiXTM software (version 9, Dental Wings GmbH, Freiburg, Germany). The outcome assessment was blinded. In all cases, the maximum possible superposition was adjusted using the automatic software function. For this purpose, the cast model was matched to at least three clearly visible matching references distributed over the arch (Fig. 2). The vestibular tooth surfaces including occlusal surfaces of the first molars as well as the distoincisal angle of the left/right central incisors were defined as references. Three-dimensional measurements (coronal, sagittal, and axial planes) included the distances between the matched files of the implant axis, implant tip and centre of the implant shoulder (Fig. 3a). Between the planned and postoperative vertical implant positions, the distances between the anterior and posterior implant shoulders and the contact of the gingiva at the implant in the sagittal planes were evaluated (Fig. 3b).

Additionally, the transfer accuracy of this study measured by postoperative CBCT scans was compared with the accuracy previously determined by using an intraoral scanner with scanbodies (angulation, lateral and vertical deviation values) [12].

### Statistical analysis

Analyses were performed using Prism 8 software for Mac OS X (GraphPad, La Jolla, California, USA). Prior to this, values were tested for normal distribution with the D’Agostino and Pearson omnibus normality test. An unpaired *t* test was used to compare implant position data, and the level of significance was set at *p* ≤ 0.05.

Post hoc power analysis was performed with the G Power software (*t* tests: difference between two independent means) to determine the power of 0.84 (angulation as a primary study aim) based on the sample size (group 1, 20; group 2, 20), using an effect size of 0.96 and an α of 0.05 (mean 1, 2.81; standard deviation 1, 2.69; mean 2, 6.22; standard deviation 2, 4.26).

## Results

**Fig. 4:**
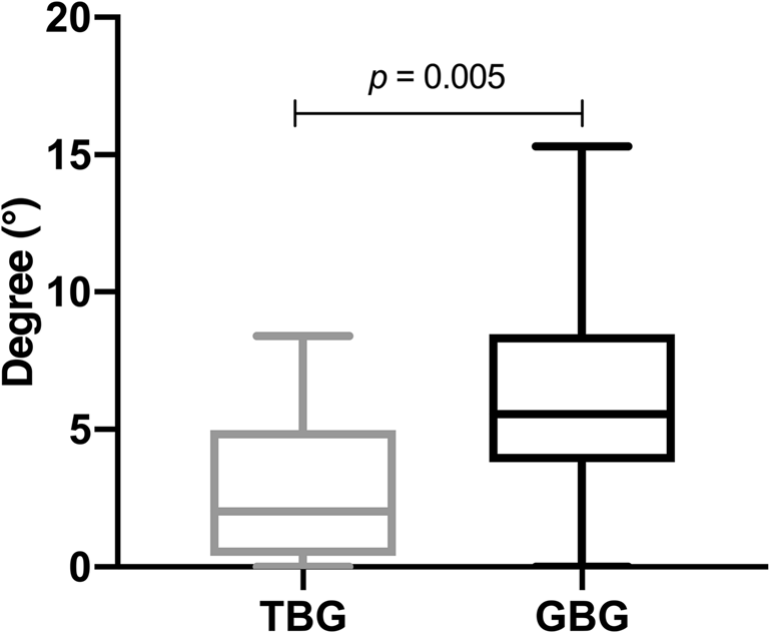
Deviation of angulation between virtual and real orthodontic mini-implant position (TBG = tooth-borne guide, GBG = gingiva-borne guide)

A significant deviation between the tooth- and gingiva-borne guides regarding the implant angulation was evaluated (*p* = 0.005, Fig. 4). The implant axis was more accurate between the two data files in TBGs (2.81°, SD 2.69) compared with GBGs (6.22° SD 4.26, Table 1).

**Table 1:** Deviations of implant axis, implant tip, centre of the implant shoulder and the vertical implant position regarding the tooth- or gingiva-borne guides with mean, standard deviation, minimum and maximum

Guide support	*N*	Parameter	Mean	SD	Min	Max	95% confidence interval
Tooth-borne	20	Angulation (°)	2.81	2.69	0.00	8.40	1.55–4.07
		Tip (mm)	1.77	0.85	0.50	3.20	1.37–2.17
		Centre of the implant shoulder (mm)	1.47	0.86	0.00	3.40	1.07–1.88
		Vertical position anterior (mm)	0.10	0.46	− 0.70	0.90	− 0.12–0.32
		Vertical position posterior (mm)	− 0.07	0.54	− 0.90	1.00	− 0.32–0.19
Gingiva-borne	20	Angulation (°)	6.22	4.26	0.00	15.30	4.23–8.21
		Tip (mm)	1.91	0.79	0.50	3.10	1.54–2.28
		Centre of the implant shoulder (mm)	1.31	0.61	0.10	2.60	1.02–1.59
		Vertical position anterior (mm)	0.22	0.58	− 1.00	1.10	− 0.05–0.49
		Vertical position posterior (mm)	− 0.31	0.66	− 1.40	0.90	− 0.61–0.01

**Fig. 5:**
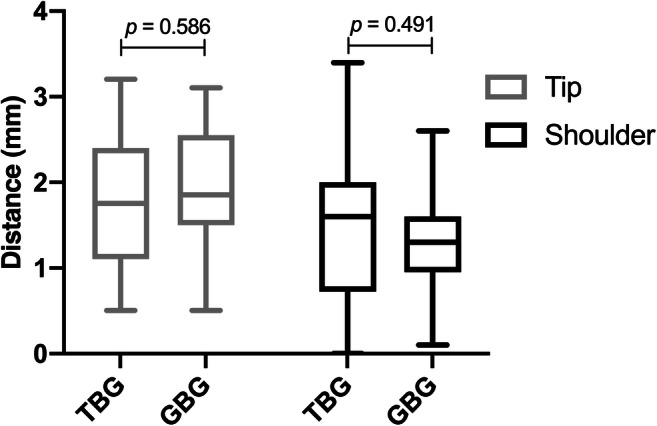
Deviation between virtual and real orthodontic mini-implant position at the tips and at the centre of each of the implant shoulders (TBG = tooth-borne guide, GBG = gingiva-borne guide)

**Fig. 6:**
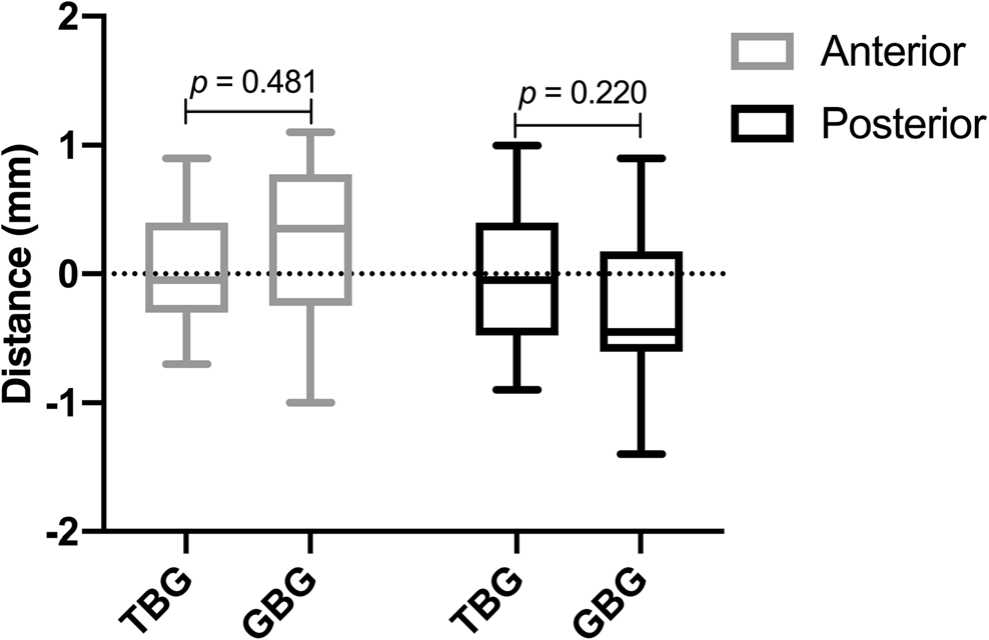
Accuracy of the vertical anterior and posterior implant shoulder positions in relation to the gingiva (TBG = tooth-borne guide, GBG = gingiva-borne guide)

Regarding all other parameters, such as the implant tip, the centre of the implant shoulder, and vertical deviations of the implant shoulders, no statistical differences were found (Figs. 5 and 6). The mean total deviations between virtual and real implant positions at the implant tips was 1.77 mm SD 0.85 for TBG and 1.91 mm SD 0.79 for GBG (Table 1). The accuracy measured at the implant tip was slightly superior in TBG; however, this was without significant differences (*p* = 0.586). On the other hand, the centre of the shoulder varied between 1.47 mm SD 0.86 (TBGs) and 1.31 mm SD 0.61 (GBGs). Furthermore, when comparing the deviations of the implant shoulder at the anterior and posterior parts, no significant difference was noted. The deviation of the implant position for all implants regarding the distance between the gingiva and the implant shoulder was positive at the anterior part (TBGs, 0.10 mm SD 0.46 vs. GBGs, 0.22 mm SD 0.58) and negative at the posterior part (TBGs, − 0.00 mm SD 0.54 vs. GBGs, − 0.31 mm SD 0.66). The highest maximum deviation values between the virtual and real implant positions were evaluated at the centre of each of the implant shoulders with a maximum deviation of 3.40 mm for TBGs and 2.60 mm for GBGs (Table 1). The highest accuracy of all parameters was investigated regarding the vertical implant positions.

**Fig. 7:**
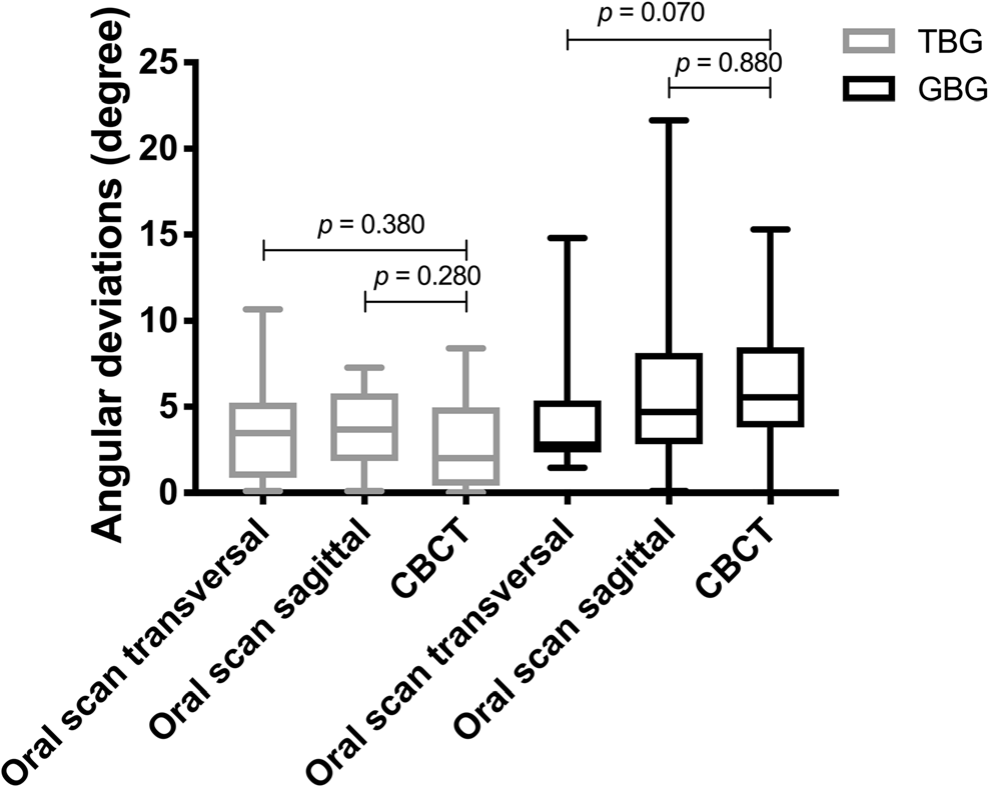
Transfer accuracy of CBCTs and oral scans in relation to the virtual planning model regarding the angulation deviations (TBG = tooth-borne guide, GBG = gingiva-borne guide)

**Fig. 8:**
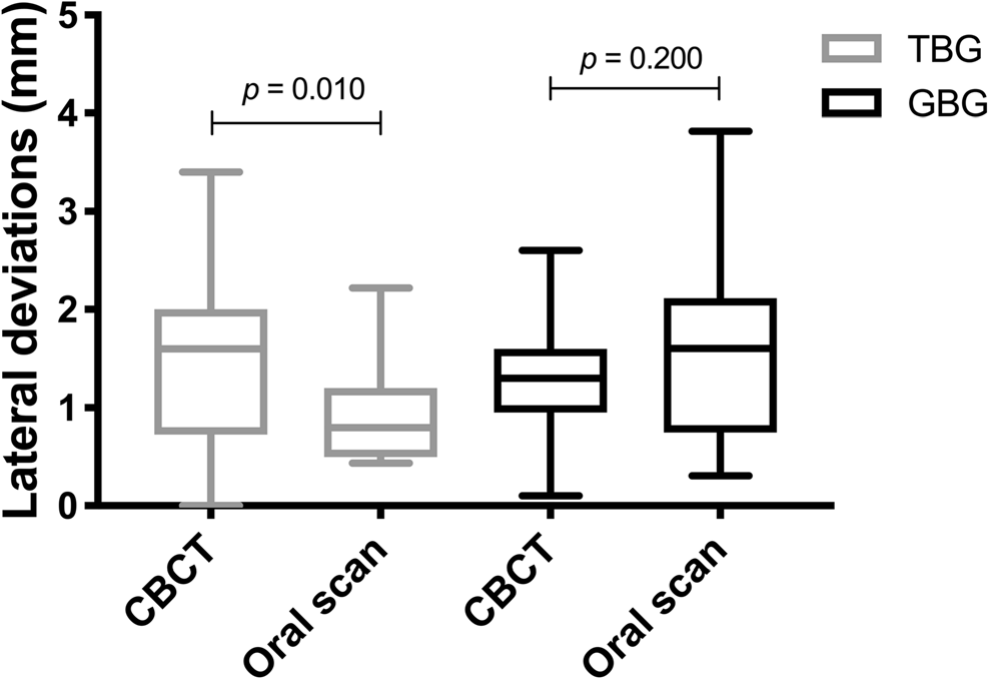
Transfer accuracy of CBCTs and oral scans in relation to the virtual planning model regarding the lateral deviations at the orthodontic mini-implant shoulder (TBG = tooth-borne guide, GBG = gingiva-borne guide)

**Fig. 9:**
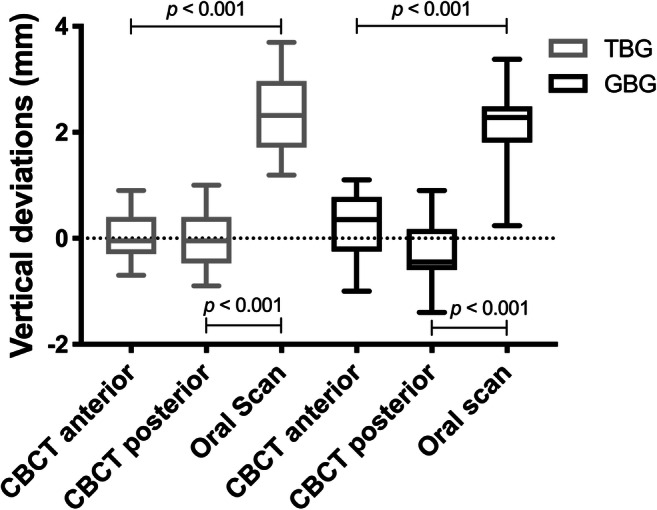
Transfer accuracy of CBCTs and oral scans in relation to the virtual planning model regarding the vertical deviations at the orthodontic mini-implant shoulder (TBG = tooth-borne guide, GBG = gingiva-borne guide)

The measurement accuracy of oral scans and CBCTs regarding the angulation deviations for TBGs and GBGs presented no significant differences, although the ranges of GBGs were much higher compared with those of TBGs, especially when using oral scans (Fig. 7). On the other hand, CBCTs of TBGs showed a significantly higher lateral deviation when compared with oral scans (Fig. 8, *p* = 0.010). Additionally, inaccuracies of oral scans were higher when compared with CBCTs of GBGs; however, these were without significant differences. Furthermore, the vertical deviation values of oral scans were significantly higher (Fig. 9; *p* < 0.001), as oral scans presented a clear inaccuracy.

## Discussion

The primary aim of this study was to assess the levels of accuracy when comparing virtual and real implant angulation. In this study, the accuracy evaluation was performed by comparing the virtual planning models and postoperative CBCT scans.

The secondary aim was to compare the transfer accuracy measured by postoperative CBCT scans with the accuracy determined using an intraoral scanner combined with scanbodies, as described in a previously published investigation [12]. Even though the use of fresh-body donors is superior to the use of fixed anatomical specimens and is closest to clinical reality [14, 15], there are some limitations in the study design that make it difficult to generalise results. On the one hand, there may be differences in tissue behaviour due the possible dehydration of bone and soft tissue as well the absence of perfusion; on the other hand, the used body donors were of advanced age, while for clinical practice, OMIs are mainly used in children. In this context, Chhatwani et al. reported that higher age is associated with a decrease in bone height in the anterior and posterior lateral palatal regions as well as the median palatal raphe [16].

Many parameters potentially determine the accuracy of fully guided implantation [17]. Template production, intraoral scanning, 3D printing, radiographical inaccuracies, matching and template fit during surgery may lead to inaccuracies during clinical applications [17–19]. Regarding the model scanning process, Koretsi et al. reported that manual model analysis by calliper and digital analysis based on a model scan by the orthoX scanner (all of which were also used in this study) were reliable within average differences of 0.5 mm for directly measured outcomes, but wide ranges are expected for some computed space parameters due to cumulative error [13]. In this context, Kernen et al. reported that accuracy can be improved if intraoral scans are used instead of surface scans of a cast model [20]. The authors concluded that an intraoral scan may reduce the cause of inaccuracies associated with a cast model. Furthermore, since the accuracy of an optical impression is similar to or better than that of an alginate impression, it may be assumed that the optical impression can be recommended for diagnostic purposes [21]. However, this statement seems to depend on the extension of the scan over the dental arch and it may therefore differ from the results of Kernen et al. regarding full arch cases. As the extension increases to cover the entire quadrant, the inferiority of the optical impressions becomes more apparent due to rising inaccuracies [21, 22]. Overall, polyether impression material showed a high level of accuracy, dimensional stability, reliability and precision in recording anatomic details [23].

In this study, vertical deviations with a range of 0.10 to − 0.07 mm and 0.22 to − 0.31 mm between virtual planning and postoperative CBCTs were more accurate than deviations between the virtual planning and postoperative oral scans (2.34-mm TBGs and 2.14-mm GBGs). Therefore, radiological and oral scan evaluations regarding levels of orthodontic OMI transfer accuracy presented significant differences.

Oral scanners from different companies presented different levels of accuracy and standard deviations during the digital impression [21, 22]. The inaccuracies of all oral scan systems will increase with the extension of the area to be captured from single tooth gaps to entire jaw sections. When scanning one jaw, as was performed in this study, a deviation tolerance of up to 378 μm might be expected.

With consideration for the importance of matching accuracy, a process with high spatial CBCT resolution is necessary to generate the best radiographic results. However, the factor that has the greatest influence is the segmentation of CBCT data, rather than the CBCT scan itself [24]. Subsequently, precise segmentation leads to higher accuracy of distinctive matching references such as teeth. These visible references (for example, ≥ 4 remaining teeth) increase the matching accuracy of model scan data with CBCT data [25]. Regarding the matching workflow of TBGs, a high accuracy and reproducibility can be expected, whereas due to missing distinctive matching references, GBGs present a more defective matching protocol.

In the context of different methods for template fabrication, Jung et al. reported no statistically significant differences among the studies [26]. In 2012, however, a systematic review in which the authors investigated the accuracy of stereo-lithographically manufactured templates in 6 in vivo studies, inaccuracies between 0.6 and 4.5 mm presented at the implant tip [24].

Also, accuracy analysis in postoperative CBCT scans represents a possible influencing parameter on the measured transfer accuracy. However, Lippold et al. reported that digital measurements obtained from virtual models created by CBCT imaging are as accurate as those taken on traditional plaster casts, which are generally assumed to accurately reflect the real situation [27].

Furthermore, OMIs in the virtual planning models were compared with those in postoperative CBCTs. However, the tooth-borne templates led to a minimal supragingival implant position measured at the anterior and posterior shoulders and to an infraposition of the implants around the GBG. A slightly insufficient fit of the TBG and the mobility of the soft tissue in the second group may explain these results. In another study, it was shown that 74% of all implants were not placed deep enough, presenting an inaccuracy when compared with the planned position [28]. According to the implant angulation, postoperative CBCT scans presented a smaller inaccuracy (2.81° TBGs versus 6.22° GBGs) with virtual planning compared with postoperative intraoral scans (3.67° TBGs versus 6.46° GBGs) [12]. However, the comparison of the deviation of the angulation can only be seen as a tendency because, in the CBCTs, both a 3D measurement and an oral scan measurement in two reference planes were performed.

## Conclusions

Within the limits of this assessment, the vertical implant position was promising in the TBG and GBG groups. The accuracy of a mini-implant position can be significantly increased by the use of a guide extended over the teeth, as previously described. However, users must keep in mind that despite virtual planning, deviations in the range of a few millimetres may occur and postoperative oral scans and CBCTs represent diverging levels of accuracy when compared with those involving virtual planning. Here, there seems to be a greater correspondence between virtual planning and direct measurement in the CBCT scans. That could mean that the transfer accuracy is higher than previously suggested by intraoral scan. Therefore, further examinations must be carried out to determine whether the precision of an indirect implant position measurement using scanbodies and an intraoral scan is sufficiently accurate in clinical use.
